# Burkitt leukaemia with B‐cell precursor immunophenotype

**DOI:** 10.1002/jha2.450

**Published:** 2022-05-04

**Authors:** Ke Xu, Anna Childerhouse, Rajeev Gupta

**Affiliations:** ^1^ Department of Haematology University College London Hospitals NHS Foundation Trust University College London London UK; ^2^ Manual Blood Sciences Health Services Laboratories University College London Hospitals NHS Foundation Trust University College London London UK; ^3^ Department of Histopathology University College London Hospitals NHS Foundation Trust University College London London UK

1

A 64‐year‐old male presented with malaise and B symptoms. An automated full blood count showed haemoglobin 124 g/L, white blood cells 40 × 10^9^/L, platelet 51 × 10^9^/L. Positron emission tomography/computed tomography indicated widespread activity above and below the diaphragm with bone marrow, peritoneal, pleural, hepatic, renal, small bowel and possible adrenal involvement.

**FIGURE 1:**
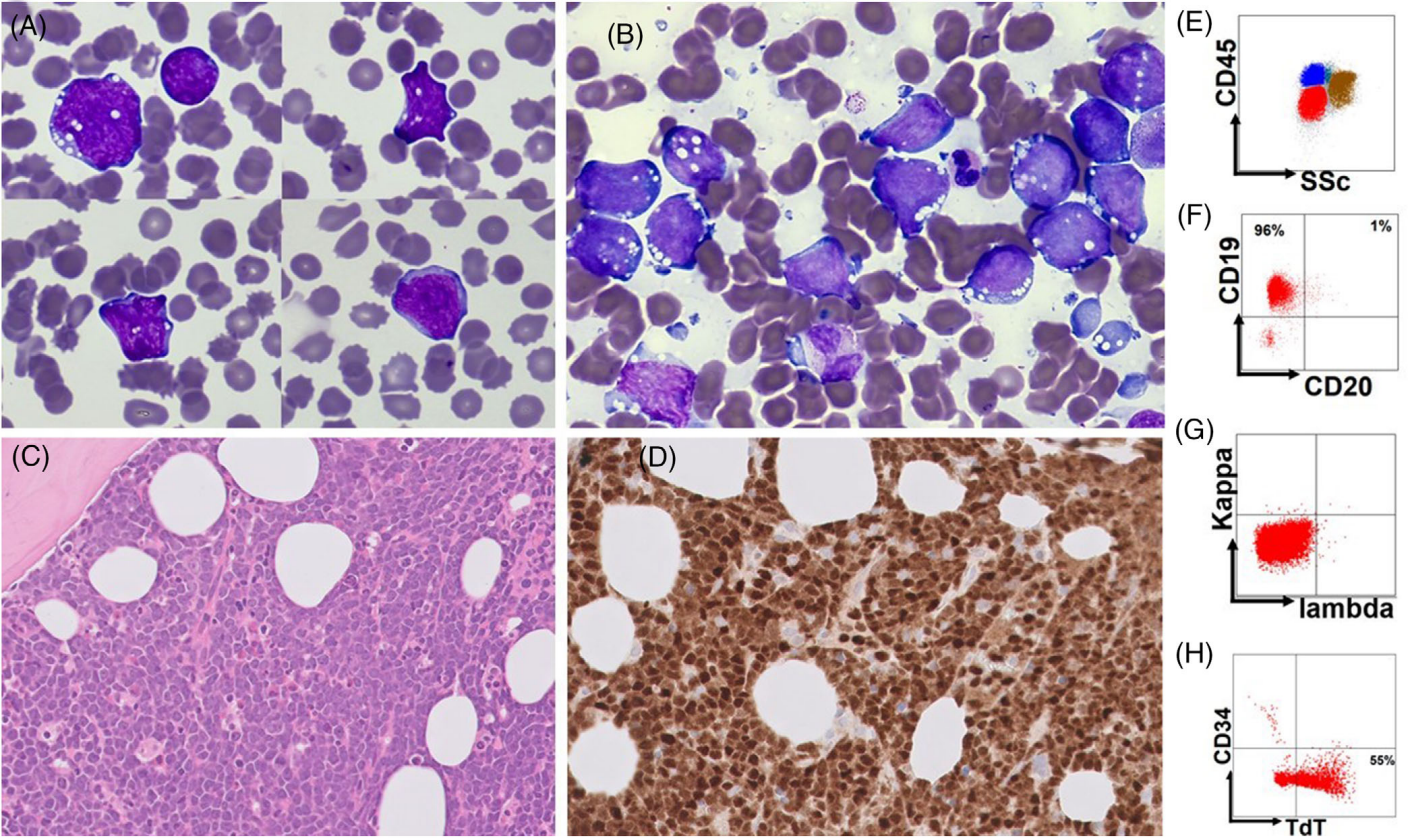
(A) Blood film (May‐Grünwald‐Giemsa stain ×100 objective); (B) Bone marrow aspirate (May‐Grünwald‐Giemsa stain ×100 objective); (C) Bone marrow trephine (haematoxylin and eosin stain ×40 objective); (D) Bone marrow trephine immunohistochemistry stainiing for TdT (×40 objective); (E–H) Immunophenotyping

The blood film (Figure 1A, May‐Grünwald‐Giemsa stain ×100 objective) showed numerous large cells with basophilic cytoplasm and cytoplasmic vacuolation. By flow cytometry (Beckman Coulter Duraclone) (Figure 1E–H) these cells (red colour population) were CD45 low (Figure 1E). They were positive for surface CD19, CD10, HLADR, CD38 and cytoplasmic CD79a, terminal deoxynucleotidyl transferase (TdT) (Figure 1H); negative for surface CD34, CD20 (panel F), CD22, light chains (Figure 1G) and cytoplasmic CD3, myeloperoxidase (MPO).

Bone marrow aspirate (Figure 1B, May‐Grünwald‐Giemsa stain x100 objective) was packed with medium to large‐sized cells with basophilic cytoplasm, and frequent cytoplasmic vacuolation. No abnormalities were detected by reverse transcription‐polymerase chain reaction screening for common leukaemic fusion genes using the Q30 leukaemia assay (QuanDX). Fluorescence in situ hybridization (FISH) analysis of the liquid sample detected immunoglobulin heavy chain/*MYC* translocation and gain of 1q. Next‐generation sequencing (Archer VariantPlex) identified *NRAS* p.Gln61Arg variant (VAF 36%).

Bone marrow histology (Figure 1C, haematoxylin and eosin stain x40 objective) revealed effacement by large cells with finely dispersed chromatin, with frequent mitoses and some with multiple small nucleoli. A classical ‘starry sky’ pattern was not seen. Immunohistochemical staining was strongly positive for CD45, CD20 (most positive) and TdT (Figure 1D, x40 objective); positive for paired box 5 (PAX5), CD19, CD79a, CD10, multiple myeloma 1; and negative for B‐cell lymphoma 2 (BCL2), BCL6, CD5, cyclin D1, CD1a, CD99, CD117, CD34, MPO and light chains. Staining for Epstein‐Barr virus ribonucleic acid was negative. The MIB1 proliferation factor was near 100%. FISH confirmed the *MYC* translocation but neither *BCL6* nor *BCL2* translocations were detected. A diagnosis of “Burkitt leukaemia (BL) with a B‐cell precursor immunophenotyped”. The patient's medical comorbidities made him unfit for chemotherapy and he received palliative corticosteroid treatment.

BL with precursor B cell immunophenotype is rare in adults. The WHO Classification of Tumours of Haematopoietic and Lymphoid Tissue (revised 4th edition) notes approximately 2% of paediatric cases of BL that “have a phenotype of precursor” B‐cells, with an expression of TdT, and sometimes CD34, and absence of CD20 and surface immunoglobulin expression. The reason for this aberrant phenotype remains unknown [1]. A small study reported IG‐*MYC*+ neoplasms with precursor B cell immunophenotype to resemble precursor B‐cell acute lymphoblastic leukaemia/ lymphoblastic lymphoma rather than BL in genomic, epigenomic profiling, the mutational landscape, and the DNA methylation pattern, with frequent activation of the RAS pathway [2]. Further studies are needed for a better understanding of this rare subgroup of disease (see Figure 1).
